# Advancing Spike Sorting Through Gradient‐Based Preprocessing and Nonlinear Reduction With Agglomerative Clustering

**DOI:** 10.1002/brb3.70650

**Published:** 2025-07-03

**Authors:** Mohammad Amin Lotfi, Fatemeh Zareayan Jahromy, Mohammad Reza Daliri

**Affiliations:** ^1^ Neuroscience and Neuroengineering Research Lab., Biomedical Engineering Department, School of Electrical Engineering Iran University of Science and Technology (IUST) Tehran Iran

**Keywords:** optimal features, spectral embedding, spike sorting, uniform manifold approximation and projection (UMAP)

## Abstract

**Background:**

Spike sorting is the process of separating electrical events produced by individual neurons in the nervous system, known as “spikes.” Accurate spike sorting is vital because it significantly impacts the reliability of all future analyses. Although several semi‐automated and fully automated spike‐sorting algorithms have been developed, their classification accuracy often proves insufficient. This has led researchers to resort to manual sorting, despite its time‐consuming and labor‐intensive nature. In certain conditions and for specific neuron populations, manual sorting can also be inefficient due to the presence of visually indistinguishable similarities between spikes. This underscores the necessity for the development of fully automated spike‐sorting methods capable of achieving high accuracy.

**Method:**

Unsupervised mathematical methods in spike sorting possess an advantage over supervised machine learning and deep learning models as they require no training and involve lower computational costs. The spike‐sorting methodology comprises two key steps: data preprocessing and spike classification. In this proposed method, a mathematical technique for data preprocessing is introduced, and nonlinear transformations are incorporated to optimally extract features from spike waveforms. The objective is to extract highly informative features that effectively separate clusters by harnessing advanced transforms, specifically uniform manifold approximation and projection (UMAP) and spectral embedding. The feature extraction process is centered around capturing inherent variations in spike waveforms, assuming that strong signal correlations enable the extraction of optimal features. Finally, a density‐based clustering algorithm is employed for spike sorting.

**Results:**

On Dataset1, GSA‐Spike and GUA‐Spike attained 100% accuracy for non‐overlapping spikes and 99.47% (GSA‐Spike) and 99.21% (GUA‐Spike) accuracy for overlapping spikes on the same dataset. In the challenging portion of the dataset, our models demonstrated a 12% improvement in accuracy. Furthermore, in the synthetic data, the efficacy of our proposed models was evident in both unit detection and spike clustering.

**Conclusion:**

The findings of our research demonstrate unparalleled accuracy, surpassing the performance of other state‐of‐the‐art methods.

## Graphical abstract

We propose an unsupervised spike‐sorting framework combining gradient‐based waveform preprocessing with nonlinear dimensionality reduction (UMAP/spectral embedding) and hierarchical agglomerative clustering. Our method (**GSA‐Spike** and **GUA‐Spike**) extracts noise‐robust features via *n*th‐order temporal derivatives, projects data into optimized low‐dimensional manifolds to resolve overlapping spikes, and clusters using silhouette‐guided merging.

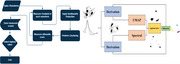

## 1 Introduction

Spike‐sorting is a critical neuroscience technique that allows the analysis of individual neural activity by matching recorded spikes to specific neural cells. To understand the underlying mechanisms of behavior and cognition, spikes are extracted and identified from extracellular recordings. Single‐unit extracellular recording involves placing electrodes in the brain tissue to record signals from a number of neurons at the same time. These recorded signals reflect changes in electrical potential caused by current flows in the extracellular medium. The data are processed using low‐pass filtering to obtain local field potential (LFP), which represents the neural dynamics near the electrode. Bandpass filtering isolates the activity of individual neurons, but overlapping spikes make it challenging to distinguish. Spike‐sorting techniques analyze spike shape characteristics, taking into account factors such as neuron morphology and distance from the electrode. Accurate spike sorting provides insight into how individual neurons respond to stimuli, their functional role in neural circuits, connectivity patterns, and the topographical organization of brain regions. It also enables the detection of sparsely firing neurons associated with higher order cognitive processes. In order to understand the complexity of brain function and cognition, spike sorting is required (Rey et al. 2015).

The aim of feature extraction methods is to capture the essence of spike data by representing it in a reduced dimensional space with concise descriptions. The use of appropriate feature extraction methods allows the data to be effectively reduced in size while retaining an adequate level of discriminative power among the extracted features (Quiroga 2012). Numerous feature extraction and dimensionality reduction algorithms have been proposed in the literature, each with its own advantages and disadvantages. In a recent study, a new method for feature extraction in neural interfaces is proposed, which can be used for spike sorting on a chip. The proposed method is based on the first and second derivative features of the spike waveform and does not require any calibration (Paraskevopoulou et al. 2013). Spike sorting often uses linear dimension reduction methods to reduce the dimension of the data while preserving relevant information. These techniques aim to preserve as much information as possible while representing the data in a lower dimensional space. They are preferred to nonlinear techniques because they're computationally efficient and easy to use. Some commonly used dimensionality reduction techniques in spike sorting include principal components analyses (PCAs) (Wold et al. 1987; Adamos et al. 2008), linear discriminant analyses (LDAs) (Keshtkaran and Yang 2017), and independent components analyses (ICAs) (Snellings et al. 2006; Takahashi et al. 2003). PCA is a widely used technique that seeks to transform the data into a set of orthogonal components that capture the maximum amount of variance in the data. In contrast, LDA aims at maximizing the separation between classes in the data by finding a linear projection maximizing the ratio of interclass variance to intraclass variance. ICA is a blind source separation technique that aims to separate the data into components that are statistically independent of each other. A recent algorithm that uses a linear dimension reduction algorithm for spike sorting, called LDA‐DP, is used and demonstrates significant results for accurate clustering of spike waveforms. The LDA‐DP algorithm (Zhang et al. 2022) integrates LDA and Dirichlet process (DP) clustering to address spike‐sorting challenges. LDA is a supervised dimensionality reduction technique that projects high‐dimensional spike waveforms into a lower dimensional subspace by maximizing the ratio of between‐class variance to within‐class variance, thereby enhancing separability of predefined neuronal clusters. However, LDA requires labeled training data to define these classes, which limits its applicability in unsupervised settings. To overcome this, LDA‐DP couples LDA with DP clustering—a non‐parametric Bayesian method that automatically infers the optimal number of clusters from the data without prior assumptions. The DP clustering component models the data using a flexible, infinite mixture of distributions, dynamically adapting to complex or overlapping spike distributions. In contrast, LDA‐GMM (Keshtkaran and Yang 2017) combines LDA with Gaussian mixture models (GMMs), which assume a fixed number of clusters, each modeled as a Gaussian distribution. Although GMM provides probabilistic assignments of spikes to clusters, its reliance on predefined cluster counts makes it less robust to datasets with variable neuronal activity. LDA‐DP outperforms LDA‐GMM in scenarios with complex or non‐Gaussian cluster geometries, as the DP's adaptive structure better accommodates overlapping or irregular spike patterns.

Spike‐sorting tasks can also use nonlinear dimension reduction algorithms. These algorithms are more complex and computationally more expensive than linear methods, but they are better able to handle the nonlinear relationships that often exist between different features in the data. Recent advancements in nonlinear dimensionality reduction have proven transformative for spike sorting, particularly in resolving overlapping waveforms and capturing subtle neural heterogeneity. A pioneering application (Nadian et al. 2018) demonstrated the power of nonlinear methods by combining *t*‐distributed stochastic neighbors embedding (*t*‐SNE) with density‐based clustering (DBSCAN) to automate spike sorting. Their approach outperformed both traditional algorithms and human experts, especially in high‐density recordings with numerous overlapping units, showcasing the potential of nonlinear techniques to disentangle complex neural activity. Extending this paradigm, Lee et al. (2021) applied *t*‐SNE to extracellular waveforms in the premotor cortex, revealing distinct cell type diversity that linear methods failed to resolve. Another relevant method is the LapEig (Laplacian Eigenmaps) algorithm (Sun et al. 2019), which employs graph‐based nonlinear dimensionality reduction to visualize neural dynamics. It constructs a similarity graph between data points and computes the eigenvectors of the graph Laplacian matrix to embed high‐dimensional data into a lower dimensional space while preserving local relationships. Although effective for capturing intrinsic manifold structures, LapEig has notable limitations: First, it is sensitive to noise and outliers due to its reliance on pairwise distances. Second, it is computational inefficient for large datasets due to its Eigen‐decomposition complexity. Third, it is limited in its preservation of global data structure, which is critical for distinguishing overlapping spike clusters (Sun et al. 2019).

Another approach for feature extraction is deep learning models such as autoencoders (AEs) (Eom et al. 2021), 1D_CNN (Li et al. 2020), and GANs (Dodon et al. 2021), which extract features by learning compact representations from data and are used in spike detection and classification tasks. Feature maps derived from neural networks can be used for various spike‐sorting problems. The ability to handle large datasets, the elimination of manual feature engineering, and the ability to learn complex patterns are advantages of deep learning feature extraction. Disadvantages include the requirement for large amounts of data and computing power, the risk of overfitting, and the inability to interpret the learned features (Rácz et al. 2020).

In this article, we propose an unsupervised model for dimension reduction and clustering. The method we propose involves preprocessing the data to ensure that the data are suitable for analyzing. Although linear dimensionality reduction techniques, such as PCA, LDA, and ICA, are computationally efficient, they assume linear relationships within the data—a limitation for spike waveforms, which often exhibit nonlinear variations due to factors such as electrode drift, noise, and overlapping neural activity. Nonlinear methods, such as uniform manifold approximation and projection (UMAP) and spectral embedding, address this problem by preserving both local and global structure in high‐dimensional data. UMAP, for example, constructs a topological representation of the data manifold, enabling robust feature extraction even in noisy or overlapping spike scenarios. Spectral embedding, on the other hand, uses graph‐based techniques to preserve pairwise distances between waveforms, making it effective for datasets with complex cluster geometries. These nonlinear methods have shown superior performance in neuroscience applications, especially where linear separability is not guaranteed (Sun et al. 2019; McInnes et al. 2018). For clustering, agglomerative clustering was selected due to its hierarchical nature and flexibility in handling clusters of varying densities and shapes—common challenges in spike data where neurons may fire at different rates or produce waveforms with subtle morphological differences. Although the algorithm ultimately requires specifying the number of clusters, its hierarchical structure allows for systematic evaluation of cluster validity using metrics like silhouette scores. This contrasts with partition‐based methods (e.g., *k*‐means), which assume spherical clusters and fixed centroids. This method has been shown to be effective in electrophysiological studies, especially when combined with nonlinear feature spaces (Herrmann et al. 2024; Campello et al. 2013).

## 2 Methods

### 2.1 Data Processing and Feature Extraction

In order to extract features that were related to the variation of the spike waveforms, we first measured the gradient of each waveform. This allowed us to capture the temporal dynamics of the spike waveform, which is an important factor in discriminating among different types of spikes when spikes are highly correlated.

To measure the *n*th gradient of each waveform, we used the first‐order central difference (Durran 1999; Quarteroni et al. 2007) of each waveform, and to obtain the *n*th gradient of each waveform, we repeated it *n* times. Central difference is a popular method to approximate derivatives in numerical analysis because it is relatively easy to calculate and provides a good balance between accuracy and computation cost:

(1) $$f^{\prime }\left(x\right)=\frac{f\left(x_{i+1}\right)-f\left(x_{i-1}\right)}{2h}+O\left(h^{2}\right)$$

where $f(x_{i})$ is the *i*th point of spike waveform, and *O* is the error of measurement. We then reduced the dimensionality of the data and removed irrelevant features by applying a nonlinearity reduction algorithm to the derivative signals. For the nonlinearity reduction step, we experimented with several different algorithms, including spectral embedding (von Luxburg 2007) and UMAP (McInnes et al. 2018).

### 2.2 Nonlinearity Reduction

**Spectral embedding** is a machine learning and data analysis technique that transforms high‐dimensional data into lower dimensional space while preserving pairwise distances among data points. This transformation is achieved by computing the eigenvectors and eigenvalues of a matrix derived from the original data. Spectral embedding represents the data as a graph, where each data point is a node and the pairwise distance between nodes is the weight of the connecting edge. From this graph, the graph Laplacian matrix is computed, a symmetric positive semidefinite matrix capturing the pairwise relationships between data points. Eigenvalues and eigenvectors of graph Laplacian are then calculated, and eigenvectors corresponding to smallest eigenvalues are used to form low‐dimensional embedding of data. Given a dataset *X* consisting of n data points, we first construct a similarity matrix **S**, where *S_ij_* measures the similarity between data points *i* and *j*. **S** can be defined in a variety of ways, but one popular way is using the Gaussian kernel:

(2) $$s_{ij}=\exp\left(\frac{-{\left\|x_{i}-x_{j}\right\|}^{2}}{2\sigma^{2}}\right)$$

where $x_{i}$ and $x_{j}$ are the *i*th and *j*th data points, ||. || represents the Euclidean distance between two points, and *σ* is a parameter that controls the width of the Gaussian. We then construct the Laplacian matrix **L**, which is defined as

(3) L=D−S

where **D** is a diagonal matrix, the diagonal entries of which are the row sums of **S**. Next, we compute the eigenvectors and eigenvalues of **L** and sort them in ascending order of their eigenvalues. Let *λ*1, *λ*2, …, *λn* be the eigenvalues, and let *v*1, *v*2, …, *vn* be the corresponding eigenvectors.

**UMAP** is a powerful nonlinear dimensionality reduction technique. It can be used to visualize and cluster high‐dimensional data. In contrast to linear techniques such as PCA (Adamos et al. 2008), UMAP (McInnes et al. 2018) is able to capture complex nonlinear relationships and is better at preserving the global structure of the data. UMAP preserves both local and global structure by constructing a low‐dimensional representation of the data. It is based on the idea of constructing a low‐dimensional representation of the data that preserves the pairwise distances between points. The algorithm works by constructing a fuzzy topological representation of the data, which is then optimized using stochastic gradient descent to minimize the cross‐entropy between the high‐ and low‐dimensional representations. The mathematics behind UMAP involves a combination of manifold theory, algebraic topology, and optimization. The algorithm constructs a fuzzy simplicial set from the data, which is a mathematical object that encodes the topological structure of the data. This is then used to construct a low‐dimensional embedding that preserves the topological structure of the data. The optimization is done using stochastic gradient descent, which involves computing gradients of the cross‐entropy loss function with respect to the low‐dimensional embedding. The gradients are computed using a combination of local and global information, which allows the algorithm to preserve both the local and global structure of the data (McInnes et al. 2018). The algorithm for UMAP can be described as follows:

1. Compute the fuzzy simplicial set representation of the data:

- For each point *i*, compute the set of *k*‐nearest neighbors *N*(*i*).
- For each pair of points *i* and *j*, compute the fuzzy set of simplices *S*(*i*,*j*) that connect them.
- Normalize the fuzzy simplicial set by setting the sum of the weights of each simplex to 1.
- Compute the fuzzy simplicial set representation of the data by taking the union of all the simplices *S*(*i*,*j*).

2 Compute the cross‐entropy between the high‐dimensional and low‐dimensional representations of the fuzzy simplicial set:

- For each pair of points *i* and *j*, compute the probability of observing the fuzzy set of simplices *S*(*i*,*j*) in the high‐dimensional representation and the low‐dimensional representation.
- Compute the cross‐entropy between the two probability distributions.

3 Compute the gradient of the cross‐entropy with respect to the low‐dimensional embedding:

- For each point *i*, compute the gradient of the cross‐entropy with respect to the low‐dimensional embedding.

4 Update the low‐dimensional embedding using stochastic gradient descent:

- For each point *i*, update the low‐dimensional embedding using the gradient computed in Step 3 and a learning rate.

5 Repeat Steps 2–4 until convergence.

Algorithm 1 | UMAP embedding

**Input**: High‐dimensional data $=\left\{x_{1},\text{…},x_{N}\right\}R^{D}$, number of neighbors *k*, minimum distance, learning rate η , epochs *T*.
**Output**: Low‐dimensional embedding $Y=\left\{y_{1},\text{…},y_{N}\right\}\subseteq R^{d}$ (where d≪D).
1. **Construct high‐dimensional fuzzy simplicial set**:

- For each $x_{i}\in X$:

a. Compute k‐nearest neighbors N(i) and distances ${\left\{d_{ij}\right\}}_{j\in N(i)}$
b. For each j∈N(i), compute membership strength:
$$\begin{array}{c} \rho_{i}=\mathrm{distance}\;\mathrm{to}\;\mathrm{nearest}\;\mathrm{neighbor},\;\sigma_{i}=\sum_{j\in N\left(i\right)}e^{-\frac{\max\left(0,d_{ij}-\rho_{i}\right)}{\sigma_{i}}}\\ \;=\log_{2}k,w_{ij}=e^{-\frac{\max\left(0,d_{ij}-\rho_{i}\right)}{\sigma_{i}}} \end{array}$$
c. Normalize weights: $w_{ij}\leftarrow w_{ij}/\sum_{j}w_{ij}$.

- Construct global fuzzy simplicial set $F=\underset{i,j}{\cup }\;S\left(i,j\right)$.

2. **Symmetrize fuzzy topology**:

- Compute probability matrix P with $p_{ij}=\left(w_{ij}+w_{ji}\right)\;-w_{ij}w_{ji}$.

3. **Initialize low‐dimensional embedding**:

- Set $Y^{\left(0\right)}=\left\{y_{1}^{\left(0\right)},\text{…},y_{N}^{\left(0\right)}\right\}$ via PCA or random initialization.

4. **Optimize embedding via cross‐entropy minimization**:

For t=1 to T:
a. Compute low‐dimensional similarities $Q^{\left(t\right)}$:
$$q_{ij}={\left(1+{\left\|y_{i}^{\left(t\right)}-y_{j}^{\left(t\right)}\right\|}^{2}\right)}^{-1}$$
b. Compute cross‐entropy loss:
$$C=\sum_{i\neq j}\left[p_{ij}\log\frac{p_{ij}}{q_{ij}}+\left(1-p_{ij}\right)\log\frac{1-p_{ij}}{1-q_{ij}}\right]$$
c. Compute gradient $\nabla_{y_{i}}C$:
$$\nabla_{yi}C=4\sum_{j\neq i}\left(p_{ij}-q_{ij}\right)\left(y_{i}-y_{j}\right){\left(1+{\left\|y_{i}-y_{j}\right\|}^{2}\right)}^{-1}$$
d. Update embeddings:
$$y_{i}^{\left(t+1\right)}\leftarrow y_{i}^{\left(t\right)}-\eta \cdot \nabla_{y_{i}}C$$
**5. Return final embedding** $Y^{\left(T\right)}$.

UMAP excels at preserving both local and global relationships within the data, making it suitable for a wide range of datasets. Nonetheless, UMAP is a relatively recent method, and its comprehensive applicability across all types of datasets is yet to be thoroughly explored. Achieving optimal performance with UMAP necessitates careful tuning of hyperparameters, including the number of neighbors and learning rate (McInnes et al. 2018). Conversely, spectral embedding is a straightforward and computationally efficient linear method. It is particularly useful when dealing with datasets that exhibit clear linear structures. Nevertheless, spectral embedding may struggle to capture nonlinear patterns in high‐dimensional data. Moreover, selecting appropriate hyperparameters, such as the number of eigenvectors to employ, can significantly impact the results obtained through spectral embedding (Belkin and Niyogi 2001).

### 2.3 Clustering

To analyze the dataset and identify meaningful patterns in this study, we used the agglomerative clustering algorithm. Agglomerative clustering is a hierarchical clustering technique. It starts with each data point as an individual cluster and iteratively merges the most similar clusters together until a stopping criterion is met.

The algorithm works as follows: (1) **Initialization**: Initially, a separate cluster is considered for each data point. (2) **Similarity calculation**: A distance metric, such as Euclidean distance or cosine similarity, is used to calculate the similarity between clusters. (3) **Merge step**: The two clusters with the highest similarity are merged into a single cluster on the basis of the calculated similarity. (4) **Update similarity**: The similarity between the new cluster and the rest of the clusters is updated in accordance with a linkage criterion, such as single linkage, full linkage, or average linkage. (5) **Repeat**: Steps 2–4 are repeated until a desired number of clusters are obtained or a stopping criterion, such as a predetermined number of iterations or a similarity threshold, is reached.

**FIGURE 1:**
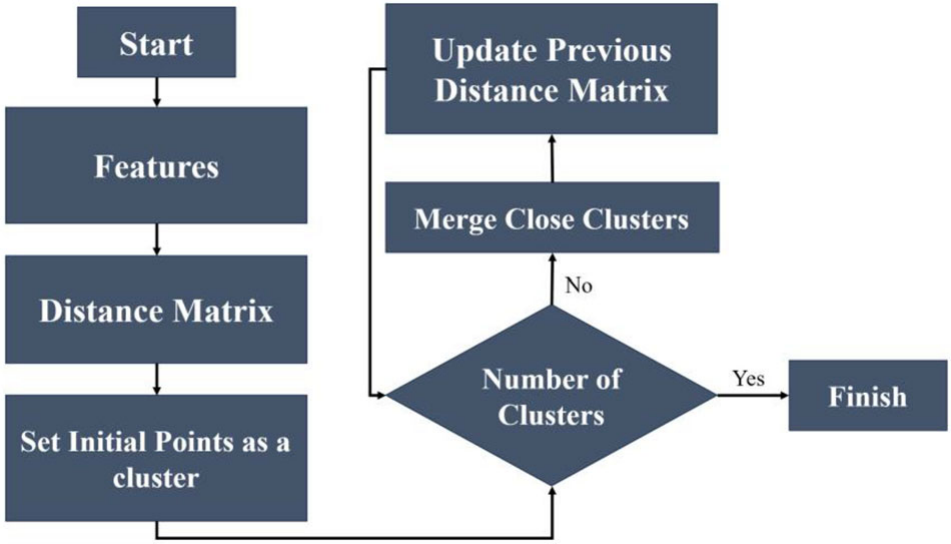
Hierarchical workflow of agglomerative clustering: block diagram illustrating the bottom‐up approach of agglomerative clustering. (1) Starting with individual data points as separate clusters. (2) Pairwise similarities are computed. (3) The most similar clusters are iteratively merged. (4) Similarities are updated until a stopping criterion (e.g., predefined cluster count) is met. The final output is a dendrogram showing hierarchical cluster relationships.

**FIGURE 2:**
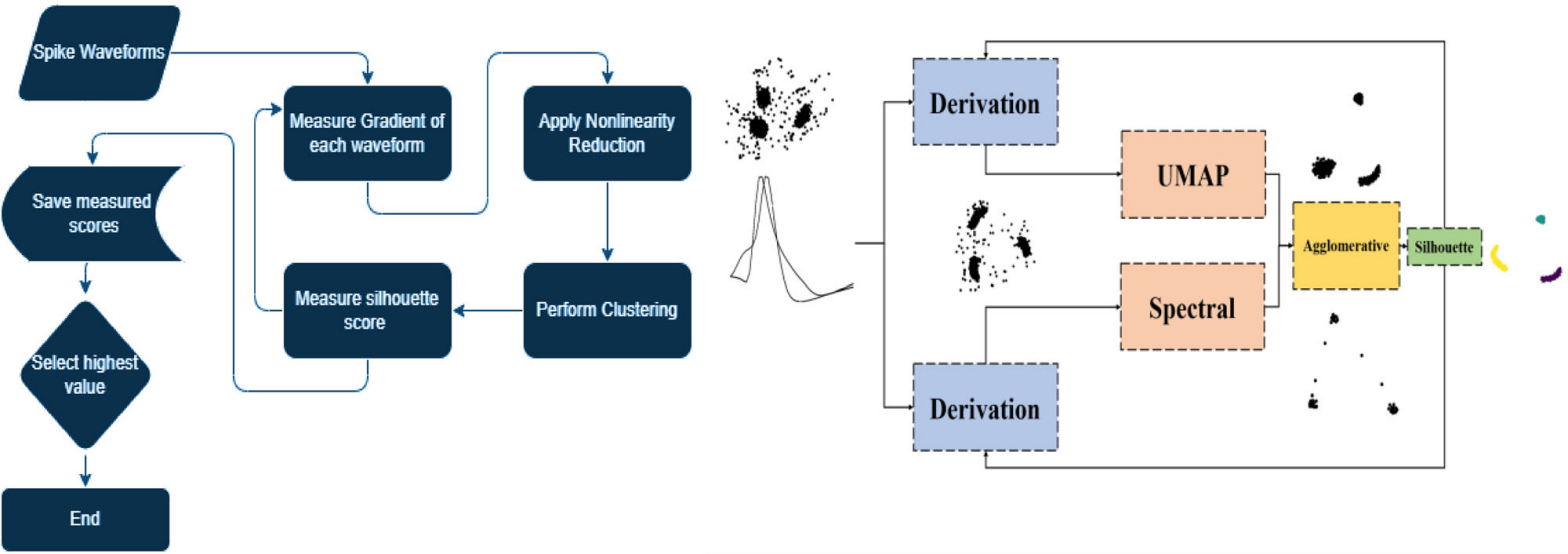
Pipeline of the proposed spike‐sorting method: flowchart of the GSA‐Spike and GUA‐Spike frameworks. (1) Raw spike waveforms undergo gradient‐based preprocessing to compute temporal derivatives. (2) Processed data are reduced via nonlinear techniques (UMAP or spectral embedding), and (3) clustered using agglomerative clustering. (4) Silhouette scores guide parameter optimization (e.g., gradient order, cluster count), and final clusters correspond to sorted neuronal units. UMAP, uniform manifold approximation and projection.

As shown in Figure 1, agglomerative clustering is known for its bottom‐up approach, whereby clusters are gradually built by merging similar data points or clusters. The result of this method is a dendrogram, which is a tree‐like structure that illustrates the hierarchy of the clusters. We used the scikit‐learn library in Python (Pedregosa et al. 2011) to implement the agglomerative clustering algorithm in our research. This library allows various clustering algorithms, including agglomerative clustering, to be implemented in an efficient and flexible way. On the basis of empirical evaluation and domain knowledge, the specific implementation parameters used in our study, such as the distance metric and linkage criterion, were chosen. We evaluated the clustering performance using the silhouette score metric by repeating this process for different orders of derivation (Equation 1), ranging from zero (zero‐order mean original data) to ninetieth order. We found that the optimal order of derivation was dependent on the specific dataset being analyzed, but in general, it ranged from first to fourth order. This suggests that the temporal dynamics of the spike waveforms are an important factor in distinguishing between different types of spikes, but that higher order derivatives do not necessarily provide additional information. Once we had identified the optimal order of derivation, we repeated the nonlinearity reduction and clustering steps using the corresponding features. The result was a set of clusters that corresponded to the different types of spikes in the dataset. We called the dimension reduction approach with spectral embedding **GSA‐Spike** and the approach with UMAP **GUA‐Spike**. All processes have been demonstrated in the flowchart (Figure 2) provided below.

Both techniques involve a variety of hyperparameters that can significantly affect the feature extraction process. In order to optimize the hyperparameters for GUA‐Spike and GSA‐Spike, a grid search method was implemented on a representative subset of the dataset. For the nonlinear projection methods (UMAP and spectral embedding), the optimal hyperparameters were determined on the basis of the best silhouette score. In a similar manner, the optimal number of clusters and the appropriate *n*th gradient order for clustering were selected by evaluating the silhouette scores across different parameter values. This methodological framework enables the adaptation of our methods to the characteristics of the dataset while providing a systematic and reproducible strategy for parameter tuning. In the case of spectral embedding, the choice of the affinity matrix can greatly affect the quality of the spectral embedding. Different types of data may require different affinities, such as the “rbf” affinity for smooth manifold structures and the “nearest_neighbors” affinity for distinct neighborhood structures. Our experiments have shown that the “nearest_neighbors” affinity is the preferred choice for spike data with complex structures, with the number of nearest neighbors automatically set to the maximum value of n_samples/10. For large datasets, the “amg” Eigen solver with a sparse affinity matrix such as “nearest_neighbors” may provide the most efficient computational approach. However, the optimal parameters will vary depending on the specific characteristics of the data. Therefore, it is crucial to explore different affinity matrices and fine‐tune their parameters to achieve high‐quality embeddings. While the use of GPU acceleration could improve performance, it is not currently available in the scikit‐learn implementation. The n_jobs parameter in scikit‐learn's spectral embedding class can control the number of parallel jobs during the computation, allowing the use of multiple CPU cores to speed up the process, especially for large datasets (Campello et al. 2013; Aeberhard et al. 1994; Herrmann and Scheipl 2020). In such cases, it is recommended to use a higher number of cores to reduce the time consumption. UMAP has some important hyperparameters that can significantly affect the quality of the embedding. The most important parameter is “n_neighbors,” which determines the trade‐off between preserving local and global structure in the data. Lower values prioritize local neighborhoods, whereas higher values emphasize the overall manifold structure. The typical range for this parameter is 5–200, with a default value of 15, although a suggested value of 50 may be more appropriate. Finding the optimal value can be time‐consuming and computationally expensive. However, the method used to find the number of clusters could potentially be used to find the optimal value for the number of neighbors, with the highest silhouette value potentially indicating the best value for the number of neighbors. The number of components indicates the dimensions to which the data is reduced in the embedding space. Higher values result in more detailed embedding but may require more computing resources. Common values for this parameter are 2 or 3, and during experiments a value of 2 was set without observing a significant impact on variation. For very large datasets (10 million points or more), the computational complexity of UMAP can become prohibitive, even with efficient implementations. If UMAP must be used on large, high‐dimensional datasets and CUDA‐enabled GPUs are available, the NVIDIA RAPIDS cuML library or the GPUMAP library are recommended options to take advantage of GPU acceleration and improve performance. However, it is important to be aware of the additional dependencies and potential portability issues compared to the CPU‐only UMAP implementation (Herrmann et al. 2024.

## 3 Dataset

We evaluated the performance of our newly developed model for clustering using two simulated datasets. The first dataset was curated by Quiroga et al. (2004) and consisted of 594 averaged spike shapes from neocortex and basal ganglia. To imitate the background noise encountered in real recordings due to the activity of distant neurons, they introduced random spikes from the database at various times and amplitudes into half of the samples. This process generated four subsets: Easy1, Easy2, Difficult1, and Difficult2, each presenting different levels of noise. The noise levels were measured in terms of standard deviation, ranging from 0.05 to 0.2 relative to the spike amplitude. In the case of Easy_1 subset, a higher noise level between 0.25 and 0.4 was deliberately introduced to facilitate the classification process. The spike amplitudes were normalized to have a peak value of 1. To mimic real recording conditions, the simulated data were initially sampled at 96 kHz and later downsampled to 24 kHz. In our research article, we referred to this synthetic dataset as “Dataset1.” To demonstrate the classification capability of our model, we specifically chose a challenging dataset where the clusters exhibited a high degree of correlation. This dataset was obtained from a study (Pedreira et al. 2012) where the authors introduced the contribution of 20 distinct spike shapes, randomly selected from a database, into the background noise to simulate multiunit activity. The firing rate of the multiunit was set to 5 Hz, and each of the 20 neurons responsible for generating the multiunit activity followed an independent Poisson distribution with a mean firing rate of 0.25 Hz. The authors employed this synthetic dataset to assess the performance of spike‐sorting algorithms under various recording conditions, with the number of source neurons varying from 2 to 20 clusters. In our study, we referred to this challenging dataset with varying numbers of source neurons as “Dataset2.” Given that real extracellular signal recordings exhibit distinctive characteristics that differentiate them from simulated data, the proposed spike detection algorithm was subjected to an evaluation on a real dataset. The dataset comprises multi‐channel recordings of both intracellular and extracellular signals from neurons in the CA1 region of the hippocampus in anesthetized rats (Henze et al. 2000). As the signals were recorded simultaneously from both the interior and exterior of the neurons, the actual spike times of one of the neuronal units can be determined from the intracellular recordings. This capability is useful for assessing the performance of the extracellular spike detection algorithm. In this study, data from the file dat16613.001 and information from Channels 2 (extracellular recordings) and 11 (extracellular recordings) were utilized.

## 4 Results

We first tested our network on Dataset1, which consists of subsets called Easy1, Easy2, Difficult1, and Difficult2. There are different levels of noise in these subsets. We compared our proposed method with several contemporary models, including DM_Kmeans (Nguyen et al. 2015), LDA_GMM (Keshtkaran and Yang 2017), Wave_Clus (Quiroga et al. 2004), LDA‐DP (Zhang et al. 2022), and an ensemble of AE (Eom et al. 2021) algorithms, in order to demonstrate the superiority of our proposed method. The ratio of correctly classified spikes to the total number of spikes in the dataset, both with and without overlapping spikes, was used to measure the accuracy of our model. Table 1 summarizes the results obtained, together with those of the comparison models. Our proposed model effectively deals with both overlapping and non‐overlapping spikes, showing reliability and achieving high classification accuracies at different noise levels. Table 1 provides a comprehensive overview of the performance comparison. The results obtained specifically for overlapping spikes are shown in brackets in the table. These results are a confirmation of the effectiveness of our approach and its ability to handle challenging spike classification scenarios. The DM_Kmeans method achieves good accuracy in discriminating different spike shapes by combining diffusion maps, silhouette statistics, and *k*‐means clustering. Compared to other spike‐sorting methods, such as superparamagnetic clustering and mean shift clustering, the proposed method using diffusion maps, silhouette statistics, and *k*‐means clustering reaches higher accuracy in distinguishing different spike shapes. Furthermore, the proposed method is unsupervised, robust, and computationally effective, which makes it a valuable tool for the analysis of large‐scale electrophysiological data (Nguyen et al. 2015). LDA_GMM proposes an unsupervised spike‐sorting algorithm. It uses discriminative subspace learning to extract more informative features from spike waveforms, resulting in improved cluster separation and higher sorting accuracy, especially for highly overlapping clusters and in the presence of noise (Keshtkaran and Yang 2017). The traditional method, Wave_Clus, is commonly used for spike sorting, but the AE deep learning model outperforms other methods in this domain. AE's capability to learn informative representations from spike waveforms makes it a compelling and powerful tool for accurate and efficient spike sorting in neuroscience research. Its optimal feature extraction performance exceeds conventional techniques, making it a promising approach in this field.

**TABLE 1:** Classification accuracy (%) on Dataset1 across noise levels: performance comparison of GSA‐Spike, GUA‐Spike, and state‐of‐the‐art methods (DM_Kmeans, LDA_GMM, Wave_Clus, LDA‐DP, AE ensemble) on Dataset1 subsets (Easy1, Easy2, Difficult1, Difficult2).

Data	Noise	DM_Kmeans (Nguyen et al. 2015)	LDA_GMM (Keshtkaran and Yang 2017)	Wave_Clus (Quiroga et al. 2004)	LDA‐DP (Zhang et al. 2022)	Ensemble of AE (Eom et al. 2021)	GSA‐Spike	GUA‐Spike
Easy1	0.05 0.1 0.15 0.2 0.25 0.3 0.35 0.4	83.09 (85.66) 91.02 (89.60) 89.98 (86.12) 95.54 (99.42) 72.91 (71.77) 71.62 (72.65) 75.65 (73.16) 87.80 (87.34)	100 100 99.81 98.17 95.66 90.79 89.3 88.43	99.96 99.81 99.81 99.55 97.52 89.5 82.12 71.98	100 100 99.9 99.4 97.7 96.4 94.9 90.7	100 100 100 99.96 100 99.44 99.63 99.63	100 (99.55) 100 (99.66) 100 (99.69) 100 (99.57) 100 (99.50) 100 (99.38) 100 (99.48) 100 (99.82)	100 (99.43) 100 (99.53) 100 (99.41) 100 (99.52) 100 (99.59) 100 (99.25) 100 (99.37) 100 (99.76)
Easy2	0.05 0.1 0.15 0.2	88.53 (90.81) 95.81 (91.60) 90.91 (92.48) 82.94 (84.98)	100 100 99.81 98.6	99.88 99.62 98.3 88.72	100 93.2 86.3 97	99.70 100 100 100	100 (99.62) 100 (99.78) 100 (99.57) 100 (99.58)	100 (99.71) 100 (99.54) 100 (99.48) 100 (99.69)
Difficult1	0.05 0.1 0.15 0.2	92.00 (87.47) 88.89 (92.79) 90.28 (86.17) 78.59 (75.98)	100 100 100 99.88	100 98.44 96.95 75.19	100 91.8 99.6 91.1	100 100 100 99.92	100 (99.56) 100 (99.83) 100 (99.63) 100 (99.88)	100 (99.61) 100 (99.87) 100 (99.63) 100 (99.79)
Difficult2	0.05 0.1 0.15 0.2	96.68 (83.95) 91.87 (94.22) 98.39 (99.01) 99.26 (98.68)	100 100 99.92 98.3	100 99.7 83.16 46.17	100 99.8 96.9 88.4	100 100 99.36 99.28	100 (99.01) 100 (98.90) 100 (98.75) 100 (98.77)	100 (98.69) 100 (98.36) 100 (97.43) 100 (96.63)
Average		88.09 (87.19)	97.93	91.32	96.2	99.81	100 (99.47)	100 (99.21)

*Note*: Values represent accuracy for non‐overlapping spikes; parentheses denote overlapping spikes. Noise levels (*σ*) range from 0.05 to 0.4 relative to spike amplitude.

The performance of the proposed model has surpassed all expectations by achieving remarkable accuracy results on Dataset1, effectively outperforming other existing methods. Specifically, for non‐overlapping spikes, the average accuracy impressively reached 100% for both GSA‐Spike and GUA‐Spike methods. Moreover, when considering the full dataset, the GSA‐Spike method attained an accuracy of 99.47%, whereas the GUA‐Spike method achieved 99.21%, thus establishing itself as the most accurate model among the comparative methods. The rigorous evaluation process entailed subjecting the model to diverse noise levels. Specifically, eight levels of noise were applied to the Easy1 subset, and four levels of noise were applied to the remaining subsets within Dataset1. Despite the varying degrees of noise, the proposed models consistently demonstrated an unwavering high level of classification accuracy. In a few specific cases, the average results from the AE and LDA‐GMM algorithms appeared similar to the outcomes of our proposed method. However, it is crucial to note that our method's consistency did not match that of the other algorithms tested. This suggests that although our approach may show similarities in average results for certain subsets, it lacks the overall robustness and reliability demonstrated by other algorithms. A further significant limitation is the inability to compare the performance of the proposed method with that of other algorithms on overlapped waveforms. This comparison was not feasible because other methods have not published their results for such scenarios, which restricts the ability to evaluate how well the proposed method handles overlapping spike waveforms, which is an essential aspect of spike‐sorting tasks. Further investigation is therefore required to fully understand the strengths and weaknesses of the proposed method compared to existing spike‐sorting algorithms. In addition to the comparison of unsupervised methods, the performance of our proposed methods has been shown to exceed that of our previous work in spike sorting. In our previous study, we developed a transformer network‐based spike denoising and sorting method that achieved an average accuracy of 99.09% on Dataset1. In the present study, GUA‐Spike and GSA‐Spike have been shown to improve the results by 0.12% and 0.38%, respectively, on the same dataset. It is important to note that although the transformer‐based model employs deep learning with self‐attention mechanisms for denoising, it necessitates a training phase and exhibits higher computational overhead. In contrast, our current fully unsupervised methods do not require any model training, thereby reducing computational complexity and rendering them more suitable for near real‐time applications. This comparison demonstrates that, although both approaches achieve high classification accuracy, the current gradient‐based methods offer practical advantages in terms of computational efficiency and ease of deployment, especially in scenarios with large‐scale electrode arrays and limited availability of labeled data.

**FIGURE 3:**
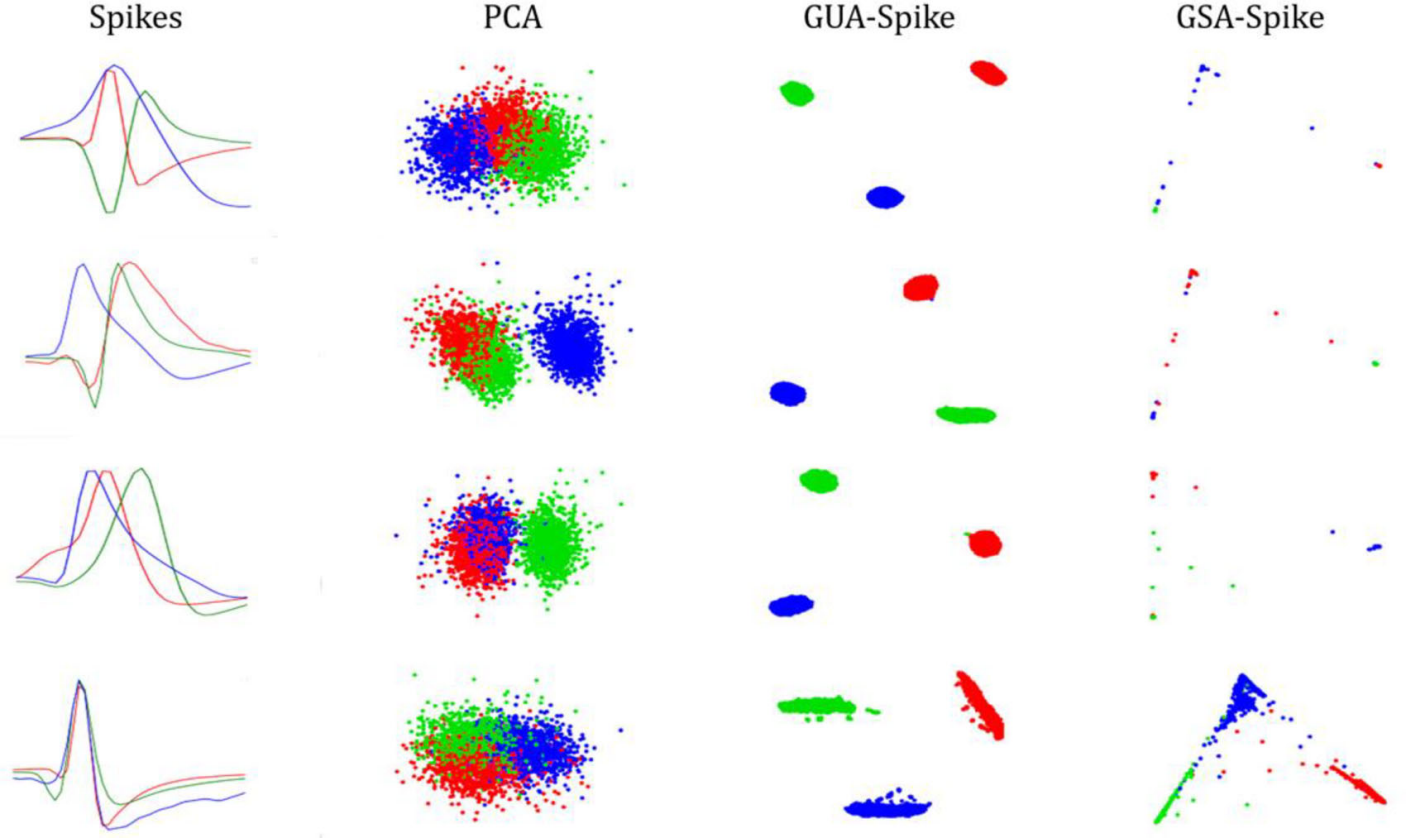
Feature subspace visualization across clustering difficulties in Dataset1: (1) easy, (2) medium, and (3) hard clustering conditions with increasing noise levels two‐dimensional UMAP embeddings of spike waveforms after gradient‐based preprocessing, color‐coded by ground truth labels. Progressive noise injection (easy to hard) illustrates degradation in cluster separability. PCA, principal components analyses; UMAP, uniform manifold approximation and projection.

To demonstrate the impact of our proposed method on the feature domain, we conducted a visualization using Figure 3. This figure presents the feature domain of all spikes before and after preprocessing and dimensional reduction. The plot showcases the original feature domain, representing the raw spike data before any modifications. Next, we illustrate the feature domain after performing gradient and dimensional reduction using our proposed technique. This reduction process condenses the data into a lower dimensional space, retaining the most relevant information while reducing noise and redundancy. By comparing the feature domains before and after applying our method, we gain valuable insights into how our approach affects the representation of spike data in the feature space. This visual analysis allows us to assess the effectiveness of our proposed method in improving the separability and clustering of spikes, providing evidence of its impact on the feature domain. Additionally, we observed that spectral embedding tends to densify the feature domain when the spikes exhibit lower correlation compared to UMAP dimensionality reduction. The plotted figures demonstrated higher silhouette scores with different orders of gradient, providing further evidence of the effectiveness of our proposed method in capturing meaningful spike clusters in the densified feature domain.

The second dataset was selected to demonstrate the efficacy of the model in enhancing the classification accuracy in the presence of a challenging task. Dataset2 comprises multiple channels, with each channel containing distinct clusters of neurons. The number of neurons in each channel varies between 2 and 20, thereby increasing the difficulty of classification as the number of neurons increases. The evaluation of the model's accuracy was conducted on channels comprising clusters ranging from 2 to 5. The analysis was exclusively focused on single‐unit activity, with deliberate exclusion of any waveforms associated with multiunit activity. This ensured that the analysis concentrated solely on the distinctive spike patterns from individual neurons, facilitating a more precise and targeted investigation of single‐unit behavior. The exclusion of multiunit waveforms served to minimize any potential interference or contamination in the data, thereby facilitating a more precise and accurate assessment of spike sorting and enhancing the overall quality of our findings.

**FIGURE 4:**
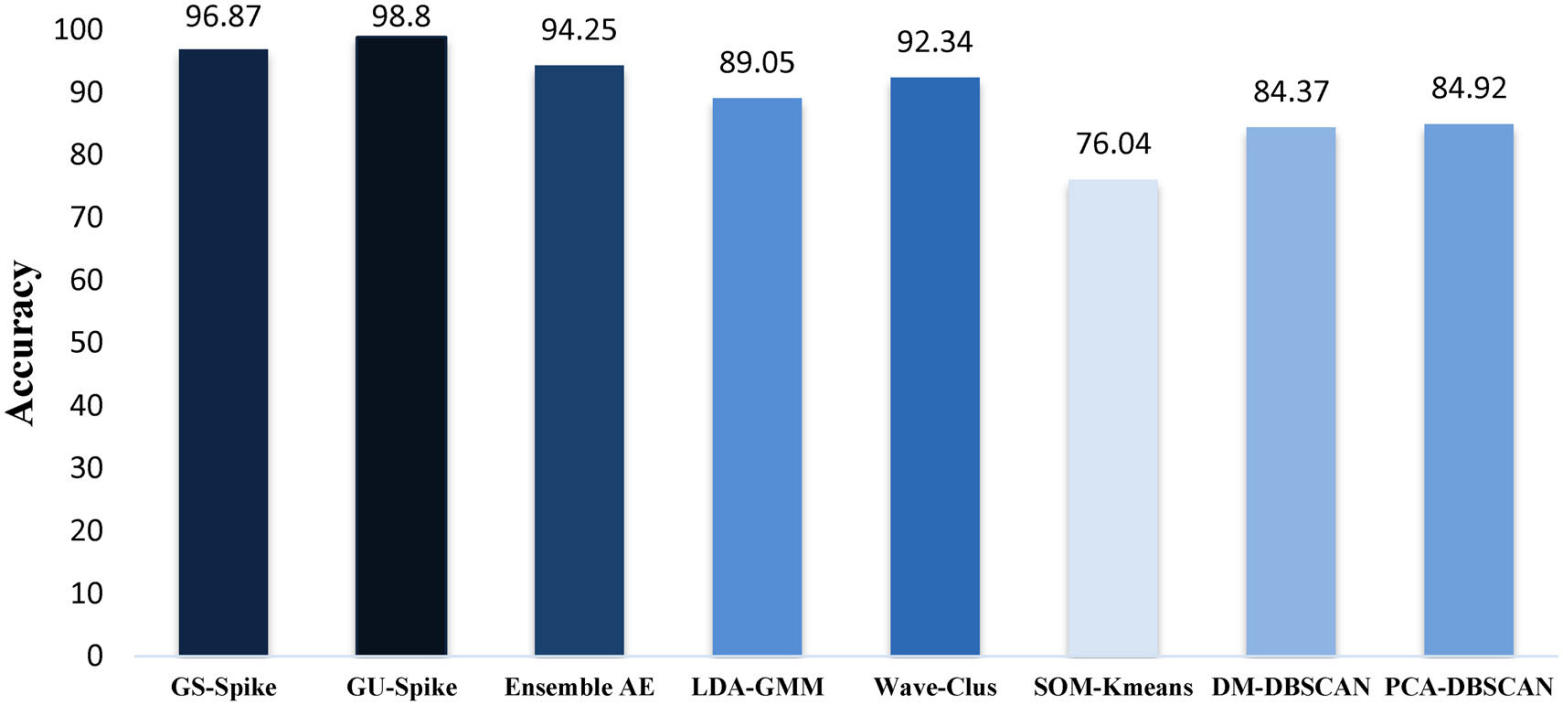
Average accuracy in Dataset2 with same number of spikes.

Tables 1, 2, 3 (Eom et al. 2021) show the performance of different spike‐sorting methods, including GSA‐Spike and GUA‐Spike, compared to other methods for different numbers of source neurons. When analyzing the results, several key observations can be made. For two source neurons, both GSA‐Spike and GUA‐Spike show excellent performance, achieving 100% accuracy across all metrics, indicating their robustness in dealing with small datasets. However, other methods such as SOM and LDA‐GMM also perform exceptionally well in this scenario, achieving 99.98% and 98.74%, respectively. As the number of source neurons increases to three and four, GSA‐Spike and GUA‐Spike maintain their superior performance, outperforming most other methods. In particular, although other methods show a slight decrease in accuracy, GSA‐Spike and GUA‐Spike remain stable and achieve 100% accuracy with four source neurons. However, when the number of source neurons reaches five, the accuracy of some methods drops significantly, whereas GSA‐Spike and GUA‐Spike still maintain high accuracy levels, demonstrating their robustness in handling larger and more complex datasets. In particular, GSA‐Spike achieves an impressive 99.80% accuracy, highlighting its effectiveness in challenging scenarios. Moreover, Figure 4 demonstrates the superiority of GU‐Spike and GS‐Spike in Dataset2, as it achieves higher average accuracy values than two state‐of‐the‐art models for spike sorting, Ensemble AE and LDA‐GM. To show how gradients make a difference, we compared our methods using two approaches: one with gradients and one without, on the dataset. Our results showed a clear difference in how we could pick out important details from the spike data when using gradients. Gradients helped us see and understand the changes in spikes much better. This highlights how important gradients are for getting accurate and useful information from spike data, and it opens up possibilities for improving how we analyze this kind of data. In simpler terms, using gradients helps us get better insights from spike data, which can improve the way we study and understand it. The names SA‐Spike and UA‐Spike are GSA‐Spike and GUA‐Spike methods, respectively, whereas they don't estimate gradient in their processes.

**TABLE 2:** Benchmarking of existing methods on Dataset2 (2–5 neurons): accuracy (%) of PCA‐DBSCAN, DM‐DBSCAN, SOM‐Kmeans, Wave_Clus, LDA‐GMM, and AE ensemble on Dataset2.

Number of neurons	No. spikes	PCA‐DBSCAN (Eom et al. 2021)	DM‐DBSCAN (Eom et al. 2021)	SOM‐Kmeans (Eom et al. 2021)	Wave_Clus (Quiroga et al. 2004)	LDA‐GMM (Keshtkaran and Yang 2017)	Ensemble of AE (Eom et al. 2021)
2	800	95.31	87.78	99.98	92.46	98.74	97.65
3	1200	89.36	92.52	85.82	93.03	86.91	94.63
4	1600	80.60	85.14	63.61	93.67	85.09	94.25
5	2000	74.42	72.04	54.77	90.21	85.49	90.49

*Note*: Spike counts increase with the number of neurons (800 spikes for 2 neurons; 2000 for 5 neurons).

**TABLE 3:** Results (accuracy %) of our proposed methods on Dataset2.

Number of neurons	Simulation number	No. spikes	GSA‐Spike	GUA‐Spike
2	8, 39, 46, 59, 94	6849	**100**	**100**
3	14, 29, 53,76, 89	7917	**100**	**100**
4	4, 21, 33, 64, 83	8924	**94.2**	**95.4**
5	30, 54, 77, 87	14,460	**93.25**	**99.80**

**FIGURE 5:**
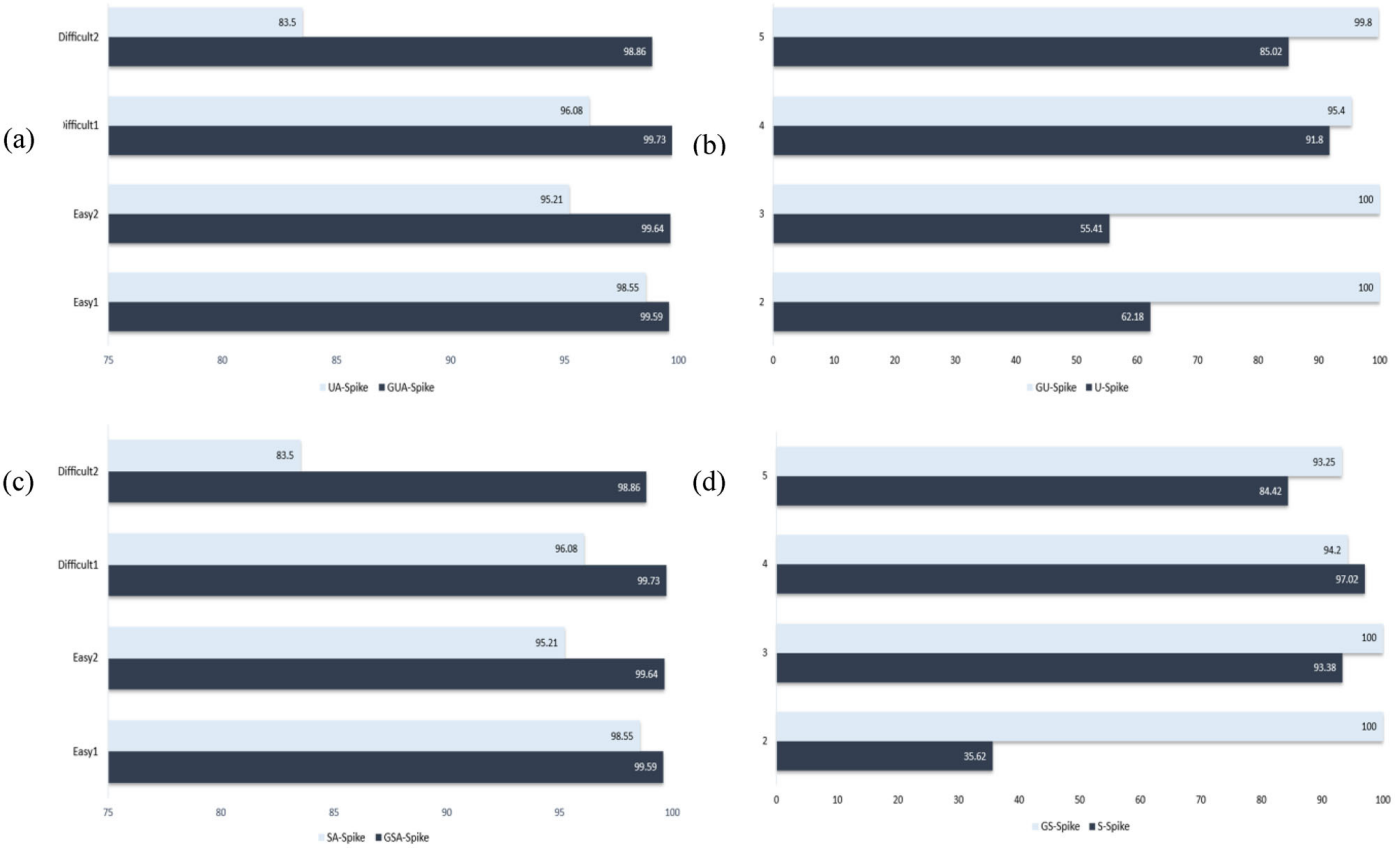
Comparison of dimensionality reduction techniques (UMAP and spectral embedding) with and without gradient‐based preprocessing on Dataset1, illustrating the impact on cluster separability: (a) UMAP dimensionality reduction without gradient preprocessing on Dataset1; (b) UMAP dimensionality reduction with gradient preprocessing on Dataset1; (c) spectral embedding dimensionality reduction without gradient preprocessing on Dataset1; (d) spectral embedding dimensionality reduction with gradient preprocessing on Dataset1. UMAP, uniform manifold approximation and projection.

The comparison shown in Figure 5 clearly illustrates a significant difference in the results. Our approach further substantiates this observation by demonstrating that the application of different gradient orders to spike data can yield more appropriate features. In essence, our method highlights the potential for improving feature extraction through the strategic implementation of different gradient orders, thereby highlighting the intrinsic information within the spikes.

The consistent accuracy and accurate prediction of the number of neurons across the two channels in Table 4 indicate that both GUA‐Spike and GSA‐Spike are effective spike‐sorting methods that can reliably identify the individual neurons in the experimental dataset. The ability to correctly estimate the number of neurons is particularly valuable because it provides important information about the underlying neural activity without the need for ground truth labels.

**TABLE 4:** Accuracy of both methods on the experimental dataset.

Data	Number of neurons	GUA‐Spike (predicted value for number of cluster)	GSA‐Spike (predicted value for number of cluster)
Channel′2′	3	97.60 (3)	98.09 (3)
Channel′11′	3	98.38 (3)	98.68 (3)

*Note*: Values in parentheses indicate the model's prediction of the optimal number of clusters.

### 4.1 Validation on High‐Density Simulated Data

**FIGURE 6:**
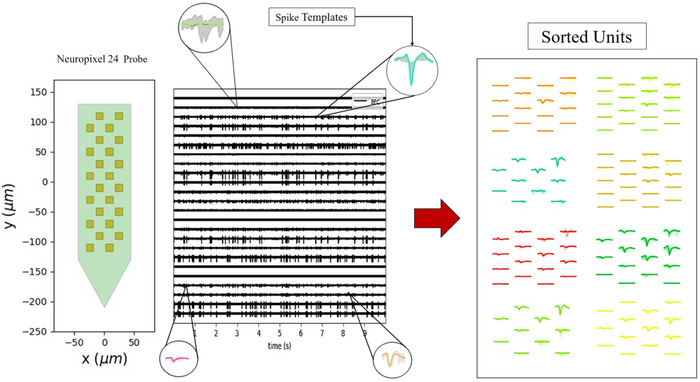
Synthesized data with a 24 Neuropixels probe with 8 units.

Spike sorting is imperative for the extraction of single‐unit activity from multi‐unit recordings, thereby facilitating a comprehensive analysis of neuronal dynamics. The development of a multi‐electrode array (HD‐MEA) (Buccino et al. 2022), among other advancements in electrode technology, has given rise to novel challenges that traditional algorithms have not yet effectively addressed. Recent spike‐sorting algorithms have been developed to address these challenges, and significant advancements have been made in this area (Lefebvre et al. 2017). To rigorously evaluate our method's scalability and robustness in modern recording paradigms, we generated synthetic high‐density datasets using MEArec (Buccino and Einevoll 2021), a Python package for simulating ground‐truth extracellular recordings with precise control over probe geometry, noise levels, and spike train dynamics. We configured a Neuropixel 24 probe (as shown in Figure 6) with 50 distinct neuronal templates (6 excitatory, 2 inhibitory units) firing at rates of 5–15 Hz over a 10‐s recording (32 kHz sampling rate, 1 µV noise). Spatial constraints (minimal inter‐unit distance: 20 µm) and amplitude ranges (100–500 µV) mimicked real‐world high‐density recording conditions. To ensure a fair comparison with existing spike‐sorting pipelines, we incorporated a spike detection step into our methodology. Spike detection was performed using a threshold‐based approach, where events exceeding 5 × *σ* (with *σ* as the standard deviation of each channel) were identified as candidate spikes. This threshold was selected to balance sensitivity and specificity, minimizing false positives while capturing true neural activity. Our spike detection achieved a precision of 99.82%, recall of 96.34%, and an *F*1 score of 98.05%, demonstrating robust performance in discriminating true spikes from noise (TP = 552, FP = 1, FN = 21). This step ensures that our pipeline no longer assumes prior knowledge of spike times, enabling equitable comparisons with methods that perform joint detection and sorting.

We compared GUA‐Spike and GSA‐Spike against state‐of‐the‐art spike sorters optimized for multi‐channel data: Herdingspikes (Hilgen et al. 2017), SpyKing Circus (Yger et al. 2018), and Klusta (Rossant et al. 2016), which can be easily implemented via SpikeInterface (Buccino et al. 2020). All methods were evaluated on the same dataset using accuracy (percentage of correctly classified spikes relative to ground truth) and unit detection rates (percentage of ground‐truth units identified).

**FIGURE 7:**
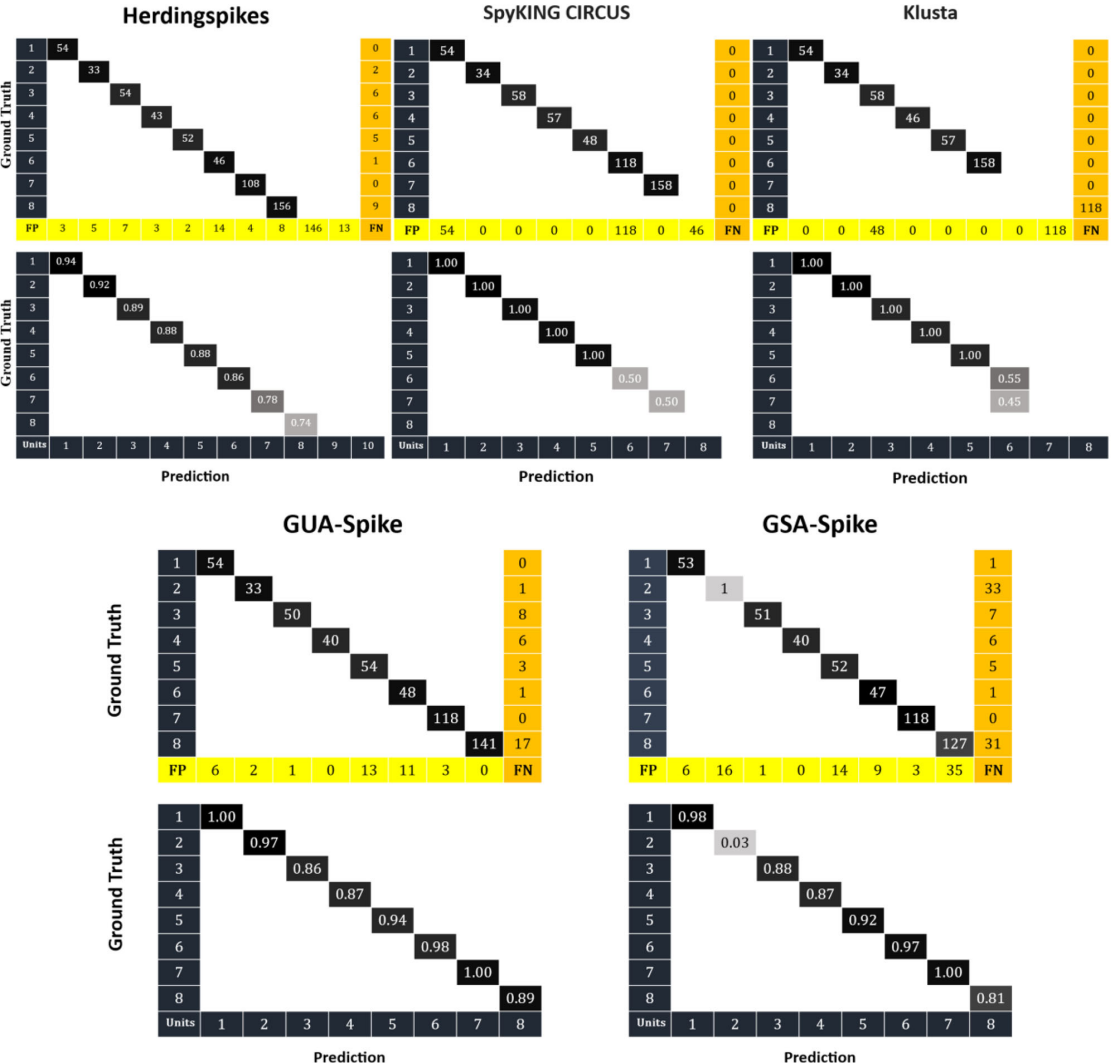
Unit detection accuracy: comparison of GUA‐Spike, GSA‐Spike, Herdingspikes, SpyKing Circus, and Klusta on synthetic 24‐channel Neuropixel data (8 units, 50 templates).

As illustrated in Figure 7, GUA‐Spike demonstrated the highest mean accuracy of 93.54%, surpassing all other methods evaluated. GSA‐Spike exhibited a commendable performance with an average accuracy of 85.23%. GUA‐Spike exhibited a high degree of accuracy in detecting all possible units, with a minimal number of false negatives, whereas GSA‐Spike, despite detecting all units, demonstrated a higher rate of false positives and false negatives, particularly in the case of unit2 spikes. Among the conventional algorithms, Herdingspikes demonstrated the highest level of accuracy at 86.61%, followed by SpyKing Circus at 75.00% and Klusta at 69.34%. The lower accuracy of Klusta suggests its reduced effectiveness in handling complex spike waveforms compared to the proposed approaches. Our methods detected Units 7 and 8 with high accuracy, whereas other algorithms either missed these units or had lower accuracy in identifying spikes of these units. These results underscore the efficacy of incorporating gradient‐based preprocessing and nonlinear dimensionality reduction in enhancing spike‐sorting accuracy.

## 5 Predict the Number of Source Neurons

**FIGURE 8:**
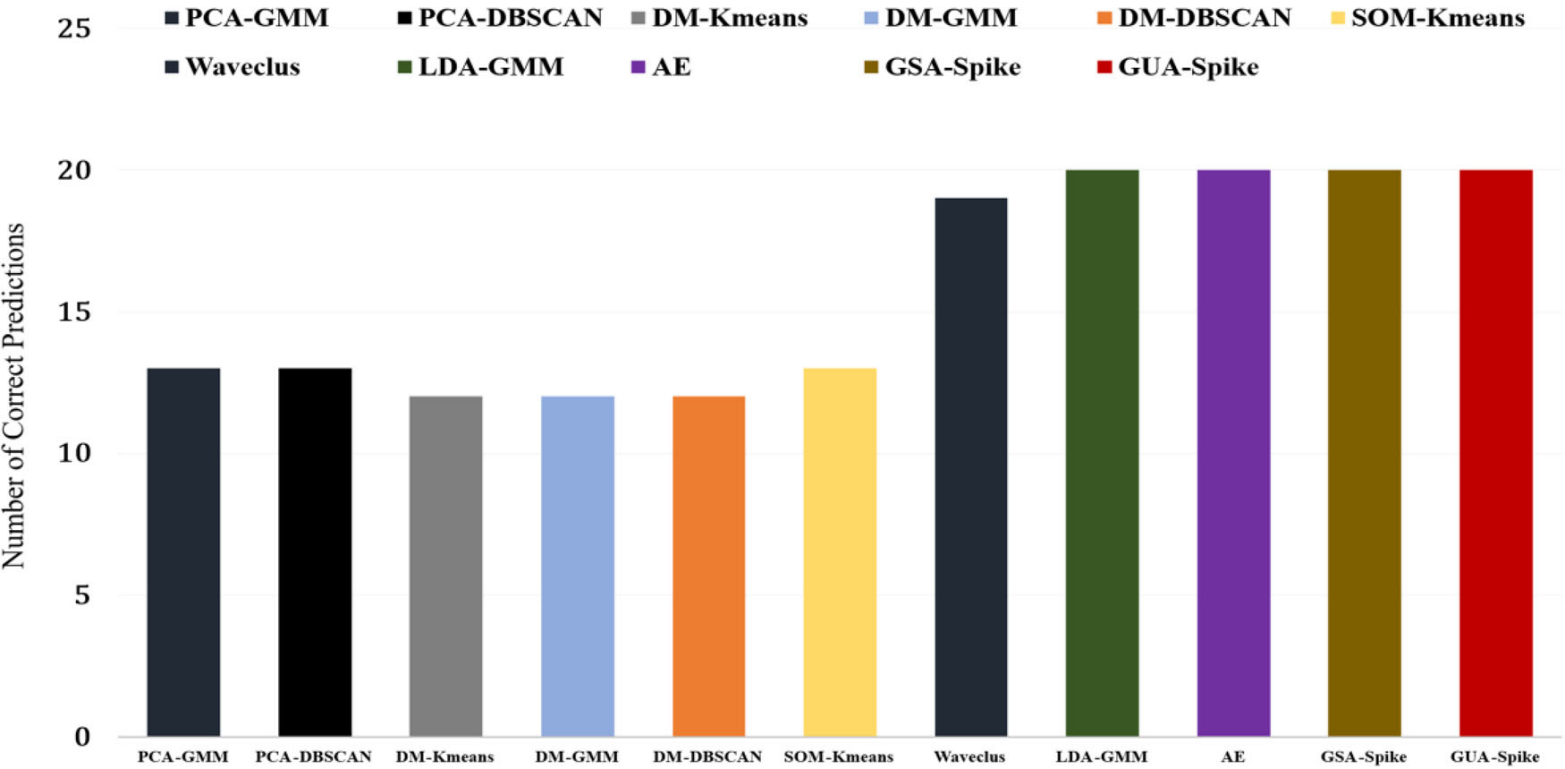
Performance of different feature extraction and clustering algorithms for the correct determination of the number of source neurons for Dataset1.

In order to predict the number of source neurons, we conducted a thorough evaluation using the silhouette score for each clustering process. We varied the number of clusters and applied the silhouette score metric to measure the quality of the resulting clusters on different channels. The silhouette score quantifies how well an individual data point fits within its assigned cluster compared to other clusters. By selecting the average highest values obtained from each clustering iteration across various channels, we aimed to identify the optimal number of source neurons that would yield the most distinct and well‐separated clusters. One of the significant challenges in spike sorting is accurately detecting the number of source neurons present in a dataset. Automating this process is crucial as it helps mitigate the potential impact of human errors on the final results. Spike sorting involves identifying and grouping spikes generated by different neurons based on their characteristic waveforms or other features. Human intervention in this process can introduce biases and inaccuracies, potentially leading to less reliable outcomes. By implementing automated methods for determining the number of source neurons, we can enhance the objectivity and consistency of spike sorting, ultimately yielding more robust and trustworthy results in neuroscientific research and analysis. Figure 8 shows the performance of the various feature extraction and clustering algorithms in correctly determining the number of source neurons in Dataset1.

The results presented in Table 5 demonstrate that GUA‐Spike exhibits superior robustness, attaining near‐perfect Silhouette scores (>95%) for two to three neurons and accurately predicting cluster counts. In contrast, GSA‐Spike exhibits erratic behavior, attaining near‐perfect scores (99.9%) for three neurons but overestimating cluster counts (e.g., predicting four instead of three). For Neurons 4 and 5, GSA‐Spike's accuracy declines significantly (Silhouette: 73%–98%), whereas GUA‐Spike maintains consistent performance (90%–95%). Both methods encounter difficulties with imbalanced data, yet they prioritize divergent metrics. GSA‐Spike places a premium on cluster separation confidence, whereas GUA‐Spike places a greater emphasis on cluster count accuracy.

**TABLE 5:** Silhouette scores (0–1 scale) of GSA‐Spike and GUA‐Spike on Dataset2, with higher scores indicating better cluster validity.

Number of source neurons	Silhouette score of GSA‐Spike	Silhouette score of GUA‐Spike	Predicted number of clusters by GSA‐Spike	Predicted number of clusters by GUA‐Spike
2	0.62	95.36	2	2
2	0.71	95.46	2	2
2	0.77	95.71	2	2
2	0.74	93.24	2	2
2	0.66	91.89	2	2
3	99.93	93.08	4	3
3	99.99	9386	4	3
3	99.99	93.28	3	3
3	99.97	93.72	3	3
3	99.99	93.55	3	3
4	97.69	94.78	4	4
4	94.74	92.11	4	4
4	79.97	90.39	4	3
4	95.37	93.97	5	4
4	92.36	94.88	4	4
5	98.44	95.33	4	5
5	79.62	92.57	5	4
5	73.58	95.87	4	5
5	99.83	95.77	4	5

**FIGURE 9:**
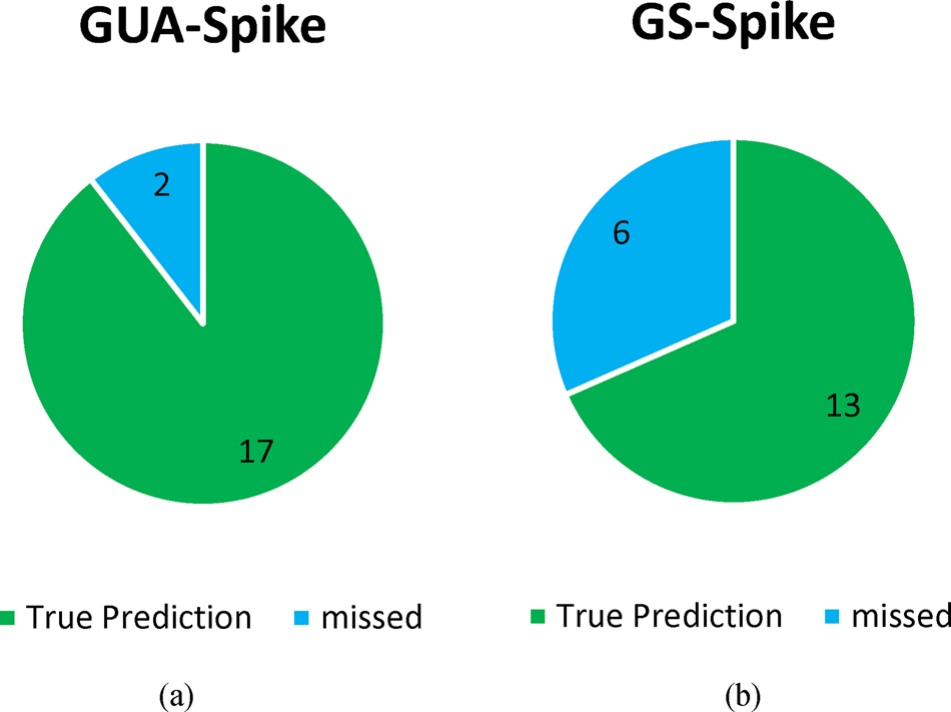
(a and b) Performance of the prediction of the number of true clusters with GUA‐Spike and GSA‐Spike, respectively, in Dataset2.

Figures 8 and 9 show that in simulated datasets, GUA‐Spike outperforms GSA‐Spike in accurately detecting the number of true clusters due to its spectral embedding feature, which has a denser data representation compared to UMAP. The higher probability of cluster mixing in spectral embedding leads to fluctuations in the silhouette score in GSA‐Spike. On the other hand, GUA‐Spike's silhouette score has less variance, making it more reliable in accurately identifying the actual number of clusters present in the data. In addition to the performance of the proposed methods on simulated datasets, they also performed well on MEA‐synthesized data. GUA‐Spike and GSA‐Spike both achieved a 100% unit detection rate, ensuring that no neuronal units were missed during sorting. In contrast, Herdingspikes (a traditional method) exhibited a 100% unit detection rate, whereas Spyking Circus (a more advanced method) demonstrated a slightly lower detection rate of 87.5%, resulting in the failure to identify one unit. Klusta exhibited the least effective unit detection performance, detecting only 75% of the units and failing to identify two neuronal units. The robustness of GUA‐Spike's results can be attributed to the enhanced clustering capabilities offered by spectral embedding, making it a superior choice for cluster detection tasks.

## 6 Discussion

**FIGURE 10:**
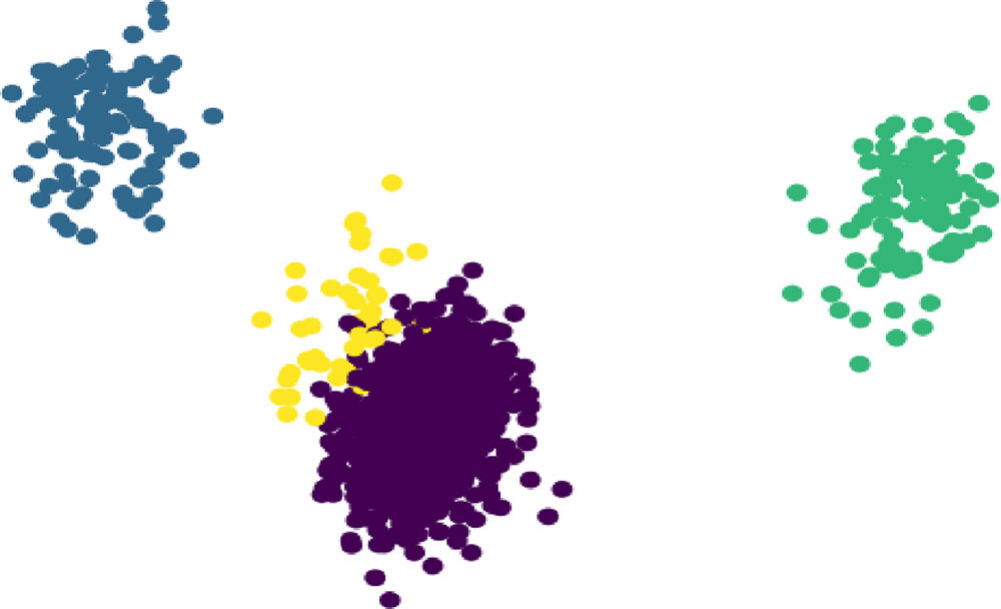
Feature domain of spikes in simulation 33.

In Section 4, the performance of our proposed unsupervised spike‐sorting algorithm was presented, including two techniques: GSA‐Spike and GUA‐Spike. We demonstrated the superiority of our models over other existing algorithms through extensive experimentation and evaluation. The selection of the best gradient order is a crucial aspect of our methods. In the majority of cases, applying the gradient resulted in a significant increase in cluster separability, leading to improved spike‐sorting accuracy. This observation was in line with our expectations, as the gradient helps in the identification of distinctive features within the data, which is conducive to better clustering. However, we encountered interesting situations where the silhouette score, a metric used to assess cluster quality, showed a decrease. An example of this occurred in the first dataset, specifically in the subset labeled “Difficult2‐015.” In this case, increasing the order of the gradient led to an increase in spike‐sorting accuracy, but at the same time led to a decrease in the silhouette score. This finding suggests that in certain scenarios, choosing the order that yields the highest accuracy may not be the optimal choice when considering cluster quality. The results of our evaluation clearly show that GUA‐Spike performs better overall than GSA‐Spike. Throughout the experiments, GUA‐Spike consistently outperformed GSA‐Spike in terms of spike‐sorting accuracy and source neuron detection. However, it is important to note that GUA‐Spike encountered a particular challenge in the second dataset, which contained four source neurons. The performance of GUA‐Spike in this dataset was affected by the presence of an unbalanced channel. Specifically, it can be seen in Table 4 that Simulation 33 presented difficulties for GUA‐Spike to accurately detect the true number of clusters, resulting in lower overall accuracy in this particular simulation. The issue of unbalanced data (like Figure 10) highlights a limitation of GUA‐Spike in certain scenarios where the data distribution deviates from the typical balanced settings. As with any algorithm, the performance of GUA‐Spike can be affected by the characteristics of the input data. In the case of unbalanced data, additional preprocessing or specific adjustments may be required to improve the accuracy and robustness of the algorithm. Despite this limitation, the superior performance of GUA‐Spike in most other cases underlines its effectiveness as a spike‐sorting algorithm.

A key advantage of our proposed spike‐sorting methods, GSA‐Spike and GUA‐Spike, is that they do not require any model training. This key feature distinguishes our algorithms from deep learning models (Eom et al. 2021; Li et al. 2020; Dodon et al. 2021; Rácz et al. 2020; Park et al. 2019; Ciecierski 2020; Liu et al. 2022; Lotfi and Daliri 2024), which often require significant time and resources to train. By eliminating the need for training, researchers are able to apply these spike‐sorting methods to their data in a simple and efficient manner, making them a sensible and practical choice for a variety of applications. We experimented to see how different parameters affected how our algorithms performed. For dimensionality reduction using spectral embedding, the results consistently showed that selecting two principal components yielded the highest performance compared to other numbers of components. This finding highlights the effectiveness of this specific parameter choice, which simplifies the implementation and allows for improved spike‐sorting accuracy. Similarly, in the case of UMAP, another dimensionality reduction technique, our experiments showed that choosing the number of neighbors between 100 and 200 was logical and effective. It was also observed that this parameter had a relatively small impact on the performance of GUA‐Spike. This consistency and insensitivity to variations in the number of neighbors further solidifies the robustness and reliability of our spike‐sorting algorithm. Our research presents strong evidence that adjusting gradient orders can play a crucial role in enhancing the performance of nonlinear dimensionality reduction algorithms, leading to improved sorting outcomes. By strategically applying various gradient orders, we have shown a substantial enhancement in accurately representing data points. This enhancement significantly benefits the overall efficacy of clustering methods. Our findings underscore the potential of manipulating gradients as a valuable technique for guiding nonlinear dimensionality reduction procedures. Ultimately, this advancement will lead to more precise and effective results in data clustering. UMAP and spectral embedding offer significant advantages over linear methods by capturing nonlinear relationships, preserving important data structures, improving clustering and visualization, and providing greater flexibility and adaptability to diverse and noisy data. In the figure below, we have compared the efficiency of feature extraction in linear and nonlinear methods.

**FIGURE 11:**
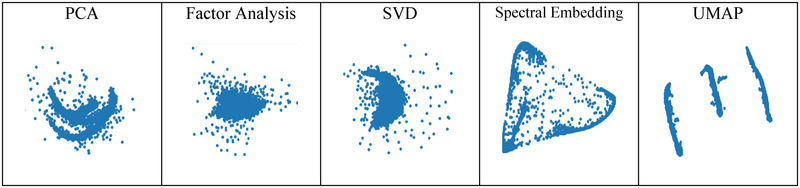
Reduced dimension of spike waveforms with different linear algorithms compared to UMAP and spectral embedding algorithms in experimental dataset—Channel′2′. PCA, principal component analysis; UMAP, uniform manifold approximation and projection.

Figure 11 illustrates the results of feature extraction using UMAP and spectral embedding applied to spike waveforms. In the UMAP plot, clusters are formed with clear separation, demonstrating the algorithm's ability to preserve both local and global structures of the data. Each cluster represents a different type of spike, demonstrating the effectiveness of the method in dealing with nonlinear relationships in the data. The spectral embedding plot also shows clear clustering, albeit with a slightly different arrangement, highlighting its efficiency in preserving pairwise distances. Linear models assume that the data lie on a linear subspace. They work by finding directions (principal components) that maximize variance (PCA), decomposing the data matrix into singular values and vectors (SVD), or modeling observed variables as linear combinations of latent factors (factor analysis). As a result, they can only capture linear relationships and cannot represent data that have a more complex, nonlinear structure. As mentioned above, UMAP works by constructing a high‐dimensional graph representation of the data, where each data point is connected to its nearest neighbors. It then optimizes the low‐dimensional representation to preserve these local neighborhood relationships while preserving the overall global structure. This balance helps UMAP preserve both small‐scale (local) and large‐scale (global) data structures. In addition, spectral embedding constructs a graph where nodes represent data points and edges represent the similarity between points. By computing the eigenvectors of the graph Laplacian, spectral embedding maps the data into a lower dimensional space while preserving the pairwise distances between points. This method is effective in preserving the overall shape and structure of the data manifold. Although some complex steps have to be applied to the data, the results of extracted features from complex spike shapes demonstrate the efficiency of the proposed nonlinear models.

The efficacy of the proposed method is evidenced by its successful application to MEArec‐simulated Neuropixel data (Buccino and Einevoll 2021), thereby underscoring its adaptability to multi‐channel recordings. Although Herdingspikes (Hilgen et al. 2017) and Klusta (Rossant et al. 2016) prioritize computational efficiency for real‐time applications, their reliance on amplitude or spatial features limits discriminability in morphologically complex scenarios. For instance, Klusta misidentified two units due to its reliance on linear PCA, which is incapable of resolving overlapping spikes with similar amplitudes but distinct temporal dynamics. Similarly, Spyking Circus (Yger et al. 2018) missed 1 unit, likely due to its threshold‐based detection struggling with low‐SNR waveforms. In contrast, GUA‐Spike's gradient‐nonlinear pipeline employs a combination of temporal dynamics and manifold learning, providing a versatile solution for contemporary, high‐channel‐count probes. This combination ensures robust unit detection and classification, even in scenarios where traditional methods falter. To evaluate computational efficiency, we benchmarked GSA‐Spike and GUA‐Spike on Google Colab using an Intel Xeon CPU @ 2.20 GHz. Tests on different dataset sizes showed different scalability patterns.

**FIGURE 12:**
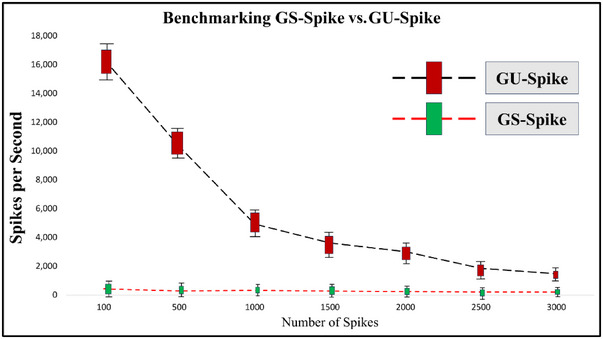
Computational efficiency benchmark: GS‐Spike versus GU‐Spike processing speed (spikes/second) versus dataset size (100–3000 spikes). GS‐Spike (green) and GU‐Spike (red) scale differently, with GU‐Spike showing superior efficiency gains at larger dataset sizes.

For smaller datasets (100–500 spikes), GS‐Spike achieved 16,395 ± 1326 to 10,314 ± 1297 spikes/s, whereas GU‐Spike processed 431 ± 113 to 277 ± 112 spikes/s. As the dataset grew to 3000 spikes, GS‐Spike maintained 1475 ± 161 spikes/s, whereas GU‐Spike dropped to 197 ± 4 spikes/s. As shown in Figure 12, GS‐Spike is faster at sorting spikes than GU‐Spike. The superior speed of GS‐Spike is due to its reliance on optimized Eigen decomposition algorithms and fixed hyperparameters, which reduce the computational overhead. In contrast, GU‐Spike's slower performance is due to its pairwise nearest neighbor graph construction and iterative optimization of nonlinear embeddings, which scale quadratically with dataset size. Although both methods exhibit reduced processing speed for larger datasets, GS‐Spike's efficiency makes it more suitable for real‐time applications, whereas GU‐Spike prioritizes accuracy in complex, nonlinearly separable clusters.

## 7 Conclusion

In summary, our novel unsupervised spike‐sorting algorithm, incorporating both GSA‐Spike and GUA‐Spike methods, demonstrated superior performance when compared to existing models. The crucial aspect of selecting the appropriate gradient order was evident, as increasing the order generally improved accuracy; however, it could have a negative impact on the silhouette score in certain cases. This emphasizes the significance of a comprehensive evaluation strategy that considers both accuracy and clustering quality to achieve effective spike sorting across diverse datasets. By leveraging advanced mathematical transformations such as UMAP and spectral embedding, our algorithms effectively extract informative features, enhancing cluster separability and enabling precise spike classification. Although the gradient‐based feature selection generally improved accuracy, it is essential to recognize scenarios where it may not optimize cluster quality optimally. Despite facing challenges with unbalanced data, GUA‐Spike consistently outperformed GSA‐Spike in various datasets, affirming its effectiveness in detecting source neurons. The key advantage of our methods lies in their training‐free nature, making them highly efficient and practical for diverse applications in neuroscience research. The robustness and reliability of our algorithms were demonstrated through extensive experiments, establishing their potential as valuable spike‐sorting tools in the neuroscience field.

## Author Contributions

**Mohammad Amin Lotfi**: methodology, software, writing – original draft, writing – review and editing. **Fatemeh Zareayan Jahromy**: writing – review and editing, supervision. **Mohammad Reza Daliri**: conceptualization, methodology, supervision, writing – review and editing.

## Conflicts of Interest

The authors declare no conflicts of interest.

## Peer Review

The peer review history for this article is available at https://publons.com/publon/10.1002/brb3.70650

## Information Sharing Statement

The following methods for implementing sorting methods and synthesizing MEA data are available at: GitHub Repository. The raw data for this study is available from the following public repositories: Leicester University: [link to dataset landing page] (Quiroga, Rodrigo Quian (2020). Simulated dataset. University of Leicester. Dataset. https://doi.org/10.25392/leicester.data.11897595.v1). Dataset2 used during the current study is available from the corresponding author on reasonable request (Pedreira et al. 2012). The experimental dataset is also publicly available and can be downloaded from https://crcns.org/data‐sets/hc/hc‐1/about (Henze et al. 2000).

## Data Availability

All the data used are from public databases and described and referenced properly in the manuscript.
